# Two New Xanthones from *Calophyllum nodusum* (Guttiferae)

**DOI:** 10.3390/molecules16118973

**Published:** 2011-10-25

**Authors:** Nadiah Mad Nasir, Mawardi Rahmani, Khozirah Shaari, Gwendoline Cheng Lian Ee, Rusea Go, Nur Kartinee Kassim, Siti Noor Kamilah Muhamad, Mohd Johadi Iskandar

**Affiliations:** 1 Department of Chemistry, Universiti Putra Malaysia, 43400 UPM, Serdang, Malaysia; 2 Department of Biology, Universiti Putra Malaysia, 43400 UPM, Serdang, Malaysia

**Keywords:** *Calophyllum nodusum*, nodusuxanthone, trapezifolixanthone A, lupeol, stigmasterol

## Abstract

The air-dried powdered stem bark of *Calophyllum nodusum* (Guttiferea) collected from Sandakan (Sabah, Malaysia), was extracted sequentially with hexane, chloroform and methanol. The solvents were removed by rotary evaporator to give dark viscous extracts. Detailed and repeated chromatographic separation of the extracts lead to isolation of two new xanthones, identified as nodusuxanthone (**1a**) and trapezifolixanthone A (**2**). Other common terpenoids such as betulinic acid, lupeol, stigmasterol and friedelin were also isolated from the extracts and identified. The structures of the compounds were established by detailed spectral analysis and comparison with previously reported data.

## 1. Introduction

*Calophyllum*, locally known as ‘bintangor’, is one of the four genus in the Guttiferae family. It consists of about 150 species found mostly in tropical Asia and the Pacific Islands. Members of the genus are usually rather slender, small to medium trees and a few are reported to have medicinal properties [1,2]. According to previous reports, the genus is a rich source of terpenoids, xanthones, coumarins, benzopyrans, and other phenolic compounds [3,4,5,6]. Some of the well-known compounds having excellent biological activity were isolated from *Calophyllum lanigerum* collected from Sarawak, Malaysia [7,8]. Dipiranotetracylic coumarins, such as (+)-calanolide A, isolated from this species, have been reported to inhibit human immunodeficiency virus type 1 (HIV-1) replication and cytophaticity, and some of them are currently being developed as chemotherapeutic agents against this virus [9,10]. In this communication we would like report the structural determination of two new xanthones, identified as nodusuxanthone (**1a**) and trapezifolixanthone A (**2**), together with four other terpenoids obtained from the crude extracts of *Calophyllum nodusum*. This is in continuation of our work on bioactive constituents of Malaysian tropical plants [11,12,13,14].

## 2. Results and Discussion

**Figure 1:**
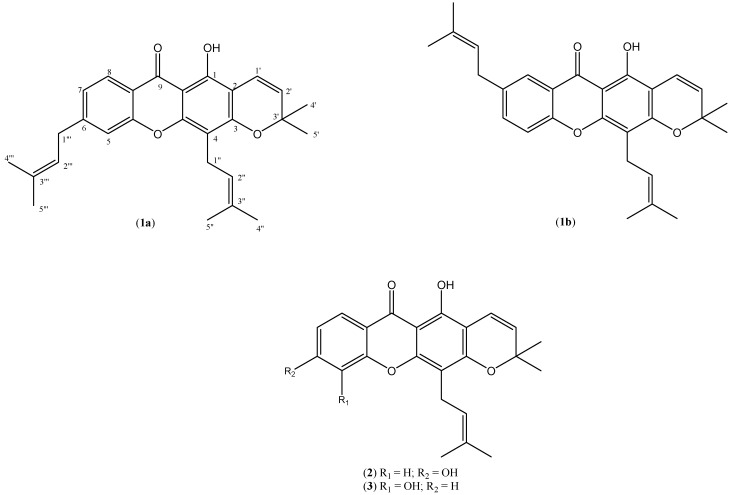
Structures of compounds **1a**, **1b**, **2**, **3**.

The air dried, powdered stem bark of *Calophyllum nodusum* (1.8 kg) was extracted at room temperature sequentially with hexane, chloroform and finally with methanol. The extracts were filtered and solvents removed by rotary evaporator to give 18.6, 31.6 and 38.1 g of dark viscous semisolid extracts, respectively. The chloroform extract was fractionated by column chromatography eluting with different mixtures of hexane, chloroform and methanol to give 168 fractions. Repeated column chromatographic separation of fractions 74-78 yielded nodusuxanthone (**1a**). Betulinic acid was also obtained from fractions 29-38 after repeated column chromatography separation. Similar fractionation of the methanol extract with vacuum column chromatography and eluting with the same solvent systems gave 118 fractions. Further separation of fractions 87-97 gave trapezifolixanthone A (**2**). Three common sterols, lupeol, stigmasterol and friedelin, were obtained from the hexane extracts by similar chromatographic separation techniques. The structures of some of the compounds are shown in Figure 1.

Compound **1a** was obtained as yellow needle-shaped crystals with m.p. 182–183 °C after recrystallisation from chloroform. The UV spectrum gave λ_max_ absorptions at 247, 284 and 305 nm, which indicated the presence of a xanthone skeleton [15] and the IR spectrum gave a strong absorption at 1,647 cm^−1^ for the chelated carbonyl group. The EIMS spectrum showed a molecular ion peak at *m/z* 430 which corresponds to the molecular formula of C_28_H_30_O_4_ with the base peak at *m/z* 323. HREIMS C_28_H_30_O_4_ gave *m/z* 430.2146 (calculated value: 430.1656). The integration of the ^1^H-NMR clearly indicated the presence of 30 protons comprising seven methines, two methylenes, six methyls and a hydroxyl (Table 1). The chelated hydroxyl group occurred at a low field region (δ 13.02). The presence of a chromene ring could be easily rationalised by the occurrence of a set of doublets at δ 5.57 and 6.09 with a common coupling constant of 10.0 Hz and a six proton singlet at δ 1.40. The aromatic A-ring is 1,2,4-trisubstitued, with the observation of two doublets at δ 7.15 (*d*, *J* = 2.3 Hz, H-5) and 7.19 (*d*, *J* = 8.0 Hz, H-8) and a doublet of doublet at δ 7.53 (*dd*, *J* = 2.3 Hz and 8.0 Hz, H-7). The COSY correlations of these methine signals further supported these assignments. The rest of the resonances were due to the presence of two sets of prenylated side chains. The signals for the two methylene groups of the side-chain integrated for four protons occurred as two doublet of doublets at δ 2.94 (*dd*, *J* = 2.7, 7.3 Hz, H-1a”, H-1a”’) and 2.96 (*dd*, 2H, *J* = 7.3, 10.8 Hz, H-1b”, H-1b”’). Similarly, the protons resonances for the two sp^2^ carbons also overlapped each other as doublet of doublet at δ 3.61 (*dd*, 2H, *J* = 2.7, 10.8 Hz, H-2”, H-2”’). While the sp^3^ methyl protons were observed as two sharp singlets each integrated for six protons at δ 1.22 and 1.16. The presence of three prenyl substituents in xanthones of *Calophyllum* species has been reported previously and in one of the compounds the prenyl substituent similarly cyclized to form a chromene ring [4,16].

**Figure 2:**
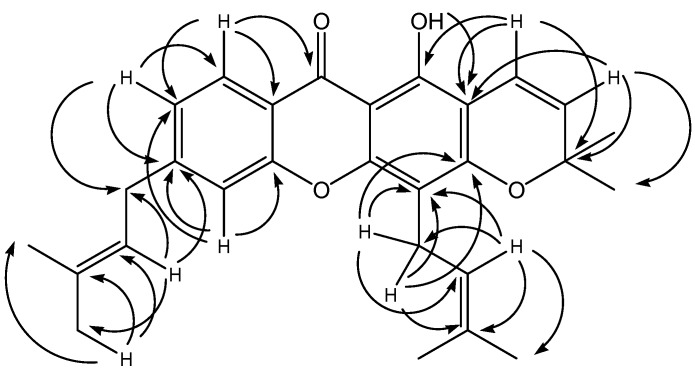
Selected HMBC correlations of **1a**.

The DEPT spectrum revealed the existence of a chelated carbonyl signal at low field (δ 182.5), seven methine, two methylene, six methyl carbons and remainder were made up of quarternary carbon atoms. Another low field signal was observed at δ 159.7 due to the hydroxyl group at C-1. The two methyl groups on the chromene ring overlapped each other and occurred at δ 28.7. The attachment of the two prenyl side-chains could be rationalised by examining the HMBC spectrum of the compound (Figure 2).

One of the side-chains is attached to C-4 of the xanthone skeleton as evidenced by the two-bond and three-bond connectivities of the methylene protons H-1” with C-3 and C-4. The attachment of the other prenyl side-chain can be either at position C-6 (**1a**) or C-7 (**1b**), since this aromatic ring is 1,2,4-trisubstituted.

**Table 1:** ^1^H-NMR and ^13^C- NMR spectral data of **1a**, **2** and **3**.

H/C	1a in CD_3_OD			2 in CDCl_3_			3 in Me_2_CO-d_6_ [17]	
	δ_H_	δ_C_	HMBC	δ_H_	δ_C_	HMBC	δ_H_	δ_C_ in
1	13.02 (*s* , OH)	159.7	C-2	13.03 (*s*, OH)	155.9	C-1, C-9a	13.33 (*s*, OH)	156.0
2		105.3			103.2			104.7
3		159.7			158.6			158.2
4		107.3			107.0			107.4
4a		155.9			153.6			153.7
5	7.15 (*d*, 2.3 Hz, 1H)	124.9	C-7, C-6, C-10a	7.26 (*d*, 1.8 Hz, 1H)	119.7	C-7, C-10a	9.22 (*s*, OH)	144.4
6		107.3		5.76 (*s*, OH)	144.4	C-5	7.38 (*dd*,1.5, 8.0 Hz)	119.7
7	7.53 (*dd*, 2.3, 8.0 Hz, 1H)	121.8	C-8, C-6, C-1”’	7.69 (*dd*, 1.8, 10.0 Hz, 1H)	116.7	C-5, C-9, C-10a	7.28 (*t*, 8.0 Hz)	123.9
8	7.19 (*d*, 8.0 Hz, 1H)	116.4	C-8a, C-9, C-7	7.19 (*d*, 10.0 Hz, 1H)	123.9	C-8a, C-10a	7.69 (*dd*, 1.5, 8.0 Hz)	116.8
8a		122.1			120.8			120.9
9		182.5			180.9			181.1
9a		104.1			104.7			103.3
10a		147.1			144.2			144.2
1’	6.09 (*d*, 10.0 Hz, 1H)	116.4	C-1, C-2, C-3’	6.70 (*d*, 10.0 Hz, 1H)	115.6	C-3’, C-9a	6.71 (*d*, 10.0 Hz)	115.7
2’	5.57 (*d*, 10.0 Hz, 1H)	128.6	C-2, C-3’, C-4’, C-5’	5.58 (*d*, 10.0 Hz, 1H)	127.4	C-3’, C-3	5.77 (*d*, 10.0 Hz)	127.4
3’		78.4			78.2			78.3
4’	1.40 (*s*, 3H)	28.7	C-2’, C-3’, C-5’	1.45 (*s*, 3H)	28.3	C-1’, C-3’, C-5’	na	28.3
5’	1.40 (*s*, 3H)	28.7	C-2’, C-3’, C-4’	1.45 (*s*, 3H	28.3	C-1’, C-3’ C4’	1.51 (*s*)	28.3
1” a	2.94 (*dd*, 2.7, 7.3 Hz, 1H)	26.0	C-3, C-4, C-2”	3.45 (*d*, 7.32 Hz, 2H)	21.7	C-4, C-2”, C-3”, C-4a	3.57 (*d*, 6.0 Hz)	21.7
1” b	2.96 (*dd*, 7.3, 10.8 Hz, 1H)		C-3, C-4, C-2”, C-3”					
2”	3.61(*dd*, 2.7, 10.8 Hz, 1H)	78.0	C-4, C-1”, C-3”, C-4”	5.18 (*t*, 7.36 Hz, 1H)	122.7	C-4”, C-5”	5.31 (*t*, 6.0 Hz)	122.6
3”		146.0			131.6			131.6
4”	1.22 (*s*, 3H)	25.5	C-2”, C-3”, C-“5	1.68 (*s*, 3H)	25.6	C-2”, 3”, C-5”	1.66 (*s*)	25.5
5”	1.16 (*s*, 3H)	30.5	C-2”, C-4”	1.83 (*s*, 3H)	17.9	C-2”, 3”, C-4”	1.87 (*s*)	17.9
1”’ a	2.94 (*dd*, 2.7, 7.3 Hz, 1H)	26.0	C-6, C-7, C-2”’					
1”’ b	2.96 (*dd*, 7.3, 10.8 Hz, 1H)		C-6, C-2”’					
2”’	3.61 (dd, 2.7, 10.8 Hz, 1H)	78.0	C-1”’, C-6, C-4”’					
3”’		146.0						
4’”	1.22 (*s*, 3H)	25.5	C-2”’, C-3”’, C-5”’					
5”’	1.16 (*s*, 3H)	30.5	C-2”’, C-4”’					

na: not available.

However, structure **1a** is more appropriate since the HMBC spectrum revealed three-bond connectivity between proton H-8 to the carbonyl C-9 and this proton also has large *ortho* coupling constant (*J* = 8.0 Hz) to H-7. Based on these spectral data, the structure of the new compound **1a** is determined as 1-hydroxy-3’,3’-dimethyl-4,6-di(3”-methyl-2”-butenyl)-2*H*,6*H*-pyrano[3,2-a]-xanthen-9-one and given the trivial name nodusuxanthone.

Compound **2** was also obtained as yellowish needle-shaped crystals with m.p. 158–160 °C. The IR spectrum exhibited a strong and broad absorption at 3,463 cm^−1^ which indicated the presence of a hydroxyl group and another strong band at 1,685 cm^−1^ assigned to a chelated carbonyl. The EIMS gave a molecular ion peak at *m/z* 378, in agreement with the molecular formula C_23_H_22_O_5_ and with a [M]^+^-CH_3_ base peak at *m/z* 363. HREIMS of C_23_H_22_O_5_ gave *m/z* 378.1467 (calculated value: 378.1452). The presence of a chromene ring in the compound was similarly rationalised in the ^1^H-NMR spectrum with the observation of a set of doublets at δ 6.70 and 5.58, each with 10.0 Hz coupling constant and an overlapped six proton singlet at δ 1.45. The protons on the aromatic A-ring also exhibited in an ABX system with the typical occurrence of two doublets at δ 7.26 (*d*, *J* = 1.8 Hz) and 7.19 (*d*, *J* = 10.0 Hz) and a doublet of doublets at δ 7.69 (*dd*, *J* = 1.8 Hz, 10.0 Hz). The xanthone skeleton is also attached to a prenyl side-chain through C-4 with the characteristic presence of signals for two methyls (δ 1.68 and 1.83), methylene (δ 3.45) and methine (δ 5.18). The ^13^C-NMR revealed the existence of 23 carbon atoms with a low field resonance at δ 180.9 assigned to the chelated carbonyl. The assignments of the proton and carbon resonances to their correct positions were further substantiated by the COSY and HMBC correlation experiments. In comparison, these data are very similar to those of trapezifolixanthone (**3**), previously reported to occur in roots of *Tovomita brevistaminea* [17] but with some very obvious differences. Thus, in compound **2** the aromatic protons in ring-A are arranged in an ABX system as can be seen in their couplings (Table 1), but in compound **3** the aromatic ring is in a 1,2,3-trisubstituted arrangement. Hence, the structure of this new compound is determined as 1,6-dihydroxy-3’,3’-dimethyl-4-(3”-methyl-2”-butenyl)-2*H*,6*H*-pyrano[3,2-a]-xanthen-9-one and it was given the name trapezifolixanthone A. Besides these two new compounds, betulinic acid, lupeol, stigmasterol and friedelin were also obtained from the hexane and chloroform extracts. Betulinic acid also occurs in other *Calophyllum* species and was reported to have antiproliferative activity against a number of cell lines which suggests its possible use as a chemotherapeutic agent [18,19]. Xanthones, especially those from *Garcinia mangostana*, are well known to exhibit strong antioxidant activity [20,21]. However, all three crude extracts and the two new xanthones failed to exhibit any free radical scavenging activity when tested with a DPPH assay (data not shown).

## 3. Experimental

### 3.1. General

Melting points were determined on a Leica Galen III apparatus. UV spectra were determined in EtOH using a Shimadzu UV-160A spectrophotometer. NMR spectra were obtained with a JEOL JNM CRX 400 MHz FT-NMR spectrometer in CDCl_3_ or CD_3_OD as solvent and tetramethylsilane as internal standard. IR spectra were obtained using a Perkin Elmer FTIR model 1725X spectrometer. EIMS were recorded on a Shimadzu GCMS-QP5050A spectrometer. Silica gel 60H 1.07736 Merck and 60 (0.063–0.200 mm) 1.07734 Merck were used for column chromatography. Precoated sheets of silica gel 60F_254_ Merck were used for TLC analysis and the spots were visualized either with a UV lamp (254 nm and 356 nm) or by iodine vapor.

### 3.2. Plant Material, Extraction and Isolation

The stem bark of *Calophyllum nodusum* was collected in October 2002 from Sabah, East Malaysia and identified by Mr. Julius Kulip of the Forest Department Sandakan, Sabah, and a voucher specimen (FRCSE 554) was deposited here. The air-dried, powdered stem bark of the plant (1.8 kg) was extracted at room temperature sequentially with hexane, chloroform and finally with methanol (5 litres each). The extracts were filtered and solvents were removed by rotary evaporator to give 18.6, 31.6 and 38.1 g of dark viscous semisolid extracts, respectively. The chloroform extract was fractionated with silica gel gravity column chromatography eluting with different mixtures of hexane, chloroform and methanol to give 168 fractions of 100 mL each. Repeated column chromatography of fractions 78-92 yielded a yellowish solid that was recrystallised from chloroform to give yellow needle-shaped crystals of nodusuxanthone (**1a**, 20 mg). Further column chromatography separation on fractions 29–38 gave betulinic acid (34 mg) as white needles, m.p. 300 °C. Similar fractionation of the methanol extract with vacuum column chromatography and elution with the same solvent systems gave 118 fractions. Further separation of fractions 87-97 yielded a yellow solid that was recrystallised from chloroform to give trapezifolixanthone A (**2**, 41 mg) as needle-shaped crystals. Three common sterols, lupeol (66 mg), stigmasterol 62 mg) and friedelin (84 mg) were also obtained from the hexane extracts by similar chromatographic separation techniques.

### 3.3. Spectral Data

*Nodusuxanthone* (**1a**). Yellow needle-shaped crystal with m.p. 182–183 °C; UV (EtOH) λ_max_ (log ε) nm: 247 (2.44), 284 (3.65), 305 (1.23); IR (MeOH) ν_max_ cm^−1^: 3,431, 2,937, 2,278, 1,720, 1,647, 1,586, 1,455, 1,374, 1,055. EIMS *m/z* (rel. int.): 430 [M]^+^ (5), 412 (32), 397 (52), 379 (8), 371 (18), 353(29), 323 (100), 309 (34), 305 (38), 302 (15), 281 (14), 269 (25), 189 (18), 59 (62); HREIMS C_28_H_30_O_4_ gave *m/z* 430.2146 (calculated value: 430.1656); ^1^H-NMR (CD_3_OD; 400 MHz) and ^13^C-NMR (CD_3_OD; 100 MHz), see Table 1.

*Trapezifolixanthone A* (**2**). Needle-shaped cryatals with m.p. 158–160 °C; UV (EtOH) λ_max_ (log ε) nm: 272 (1.61), 293 (2.55); IR (MeOH) ν_max_ cm^−1^: 3,463, 2,929, 2,018, 1,685, 1,645, 1,451, 1,376, 1,129. EIMS *m/z* (rel. int.): 378 [M]^+^ (30), 364 (20), 363 (100), 335 (15), 154 (17) and 55 (12); HREIMS C_23_H_22_O_5_ gave *m/z* 378.1467 (calculated value: 378.1452); ^1^H-NMR (CDCl_3_; 400 MHz) and ^13^C-NMR (CDCl_3_; 100 MHz), see Table 1.

*Betulinic acid*. White powder with m.p. 300 °C; IR v_max_ cm^−1^ (CHCl_3_): 3,698, 2,933, 1,686, 1,453, 1,373, 1,030. EIMS *m/z* (rel. int.): 456 [M^+^] (20), 248 (50), 207 (50), 189 (100). The spectral data of the compound were identical to literature values [19].

*Lupeol*. White powder with m.p. 208 °C; UV (CHCl_3_) λ_max_ (log ε) nm: 350 (0.74); IR v_max_ cm^−1^ (CHCl_3_): 3,335, 2,938, 1,452, 1,376, 1,033, 879. EIMS *m/z* (rel. int.): 426 [M^+^] (30), 218 (55), 95 (100). The spectral data of the compound are identical to literature values [20].

*Stigmasterol*. White needles with m.p. 255 °C; UV (CHCl_3_) λ_max_ (log ε) nm: 257 (1.64); IR (CHCl_3_) ν_max_ cm^−1^: 3,430, 2,934, 1,456, 1,372, 1,053, 963; EIMS *m/z* (rel. int.): 412 [M^+^] (55), 255 (43) and 55 (100). The spectral data of the compound are identical to literature values [20].

*Friedelin*. White crystals with m.p. 150 °C; UV (CHCl_3_) λ_max_ (log ε) nm: 287 (0.09); IR (CHCl_3_) ν_max_ cm^−1^: 2,927, 1,868, 1,712, 1,453, 1,384, 1,068; EIMS *m/z* (rel. int.): 426 [M^+^] (30), 273 (40), 95 (100). The spectral data of the compound are identical to literature values [21].

## 4. Conclusions

Two new xanthones have been isolated from the stem bark crude extracts of *Calophyllum nodusum*, together with the common terpenoids, betulinic acid lupeol, stigmasterol and friedelin. The new compounds were spectroscopically identified and given the trivial names nodusuxanthone and trapezifolixanthone A. Both the pure compounds and the extracts failed to exhibit any antioxidant activity when tested in the DDPH assay.
